# High sensitive C-reactive protein and serum amyloid A are inversely related to serum bilirubin: effect-modification by metabolic syndrome

**DOI:** 10.1186/1475-2840-12-166

**Published:** 2013-11-09

**Authors:** Petronella E Deetman, Stephan JL Bakker, Robin PF Dullaart

**Affiliations:** 1 Department of Nephrology, University of Groningen, University Medical Center Groningen, Groningen, The Netherlands; 2 Department of Endocrinology, University of Groningen, University Medical Center Groningen, P.O. Box 30.001, Groningen 9700 RB, The Netherlands

**Keywords:** Metabolic syndrome, Serum amyloid A, Bilirubin

## Abstract

**Background:**

Bilirubin has been implicated in cardiovascular protection by virtue of its anti-inflammatory and anti-oxidative properties. The metabolic syndrome is featured by enhanced low-grade systemic inflammation and oxidative stress. Serum amyloid A (SAA) impairs anti-oxidative properties of high-density lipoprotein (HDL). We determined relationships of high sensitive C-reactive protein (hs-CRP) and SAA with bilirubin in subjects with and without metabolic syndrome (MetS).

**Methods:**

Serum total bilirubin, hs-CRP, SAA and homeostasis model assessment- insulin resistance (HOMA-IR) were documented in 94 subjects with and in 73 subjects without MetS (26 and 54 subjects with type 2 diabetes mellitus (T2DM), respectively).

**Results:**

Bilirubin was lower in MetS (*P* = 0.013), coinciding with higher hs-CRP (*P* < 0.001) and SAA levels (*P* = 0.002). In all subjects combined, hs-CRP was inversely related to bilirubin (r = −0.203, *P* = 0.008), irrespective of the presence of MetS or T2DM (interaction terms: *P* ≥ 0.75). The inverse relationship of bilirubin with SAA was confined to subjects without MetS (r = −0.267, *P* = 0.009). Furthermore, the presence of either MetS or T2DM modified the relationship of bilirubin with SAA (interaction terms: β = 0.366, *P* = 0.003 and β = 0.289, *P* = 0.025, respectively) in age- and sex-adjusted analyses. Effect modification was also found for HOMA-IR (β = 0.790, *P* = 0.020). Of the individual MetS components, the strongest interaction of bilirubin on SAA was observed with low HDL cholesterol (β = 0.435, *P* < 0.001).

**Conclusions:**

hs-CRP is inversely related to bilirubin, suggesting that low bilirubin is implicated in enhanced low-grade systemic inflammation. The inverse relationship of SAA with bilirubin was found to be absent in MetS, which could be attributable to MetS-associated abnormalities in HDL characteristics.

## Background

Bilirubin has well known anti-oxidative and anti-inflammatory properties, as evidenced by its ability to scavenge peroxyl radicals, to inhibit low density lipoprotein (LDL) oxidation and to downregulate expression of the cellular adhesion molecules, VCAM-1 and ICAM-1 *in vitro*[1,2]. During the past few years the concept is emerging that bilirubin is involved in the pathogenesis of several disorders featured by enhanced systemic low-grade inflammation and increased oxidative stress, such as atherosclerotic cardiovascular disease, metabolic syndrome (MetS) and Type 2 diabetes mellitus (T2DM) [3-8]. Accordingly, isolated hyperbilirubinemia confers decreased intima media thickness, a marker of subclinical atherosclerosis [9,10]. Conversely, low serum bilirubin levels determine increased intima media thickness [11], increased atherosclerosis severity [12] and higher risk of lower limb amputation in T2DM [13]. High bilirubin levels were also documented to predict low cardiovascular and all-cause mortality in men [14].

In agreement with the notion that higher bilirubin levels may contribute to reduced systemic low-grade inflammation, some studies have shown inverse relationships of high sensitive C-reactive protein (hs-CRP) with serum bilirubin [11,15,16]. Little information is, however, available concerning relationships with other inflammatory markers [17]. The pro-inflammatory protein, serum amyloid A (SAA), is able to impair anti-oxidative properties of high density lipoprotein (HDL) [18-20], thereby contributing to increased oxidative stress. Both hs-CRP and SAA are established predictors of incident cardiovascular disease [21-23]. Furthermore, the metabolic syndrome is not only characterized by lower serum bilirubin, but also by higher hs-CRP and SAA levels [18,24-26]. In view of the anti-inflammatory and anti-oxidative properties of bilirubin, as well as lower bilirubin levels together with higher hs-CRP and SAA levels in MetS, we decided to test the extent to which possible relationships of bilirubin with hs-CRP and SAA are modified by the presence of MetS.

We therefore initiated the present study to establish relationships of hs-CRP and SAA with bilirubin in subjects with and without MetS.

## Methods

### Subjects

The medical ethics committee of the University Medical Center Groningen, The Netherlands approved the study and all participants provided written informed consent. This study was performed in a university hospital setting. The subjects (aged > 18 years) were Caucasian, and were recruited by advertisement in local newspapers. In total, 167 subjects were included. MetS was defined according to the revised National Cholesterol Education Program-Adult Treatment Panel III (NCEP-ATP III) criteria [27]. Three or more of the following criteria were required for categorization of subjects with MetS: waist circumference > 102 cm for men and > 88 cm for women; blood pressure ≥ 130⁄85 mmHg or use of anti-hypertensive drugs; fasting plasma triglycerides ≥ 1.70 mmol/l; HDL cholesterol < 1.0 mmol/l for men and < 1.3 mmol/l for women; fasting glucose ≥ 5.6 mmol/l. Subjects with Type 2 diabetes mellitus (T2DM), previously diagnosed by primary care physicians using guidelines from the Dutch College of General Practitioners (fasting plasma glucose ≥ 7.0 mmol/l and/or non-fasting plasma glucose ≥11.1 mmol/l) were allowed to participate. Diabetic subjects were treated by primary care physicians with diet alone or diet in combination with metformin and/or sulfonylurea. The use of anti-hypertensive medication was allowed, but the use of insulin was an exclusion criterion. Further exclusion criteria were clinically manifest cardiovascular disease, current smoking, renal insufficiency (elevated serum creatinine and/or proteinuria), thyroid disorders, liver disease, pregnancy, and use of lipid lowering drugs. Physical examination did not reveal pulmonary or cardiac abnormalities. All subjects were studied after an overnight fast. Body mass index (BMI) was calculated as weight divided by height squared (in kg/m^2^). Waist circumference was measured between the 10th rib and the iliac crest. Insulin resistance was estimated using the Homeostasis Model Assessment-Insulin Resistance (HOMA-IR): glucose (mmol/l) × insulin (mU/l) / 22.5. Alcohol consumption was estimated with one drink being assumed to contain 10 grams of alcohol.

### Laboratory analyses

Venous blood samples were collected into EDTA-containing tubes (1.5 mg⁄ml) for measurement of SAA, hs-CRP and lipids. Serum was obtained for measurement of total bilirubin and transaminases. Plasma and serum samples were prepared by centrifugation at 1400 g for 15 min at 4°C. Blood glucose and glycated hemoglobin (HbA1c) levels were measured directly after blood collection. Samples for other assays were stored at −80°C until analysis.

Serum total bilirubin was measured by colorimetric assay with a detection limit of 1.0 μmol/l (2,4-dichloroaniline reaction; Merck MEGA, Darmstadt, Germany). The inter-assay coefficients of variation amounts to 3.8% and 2.9% in the lower normal and higher normal range respectively. In healthy subjects, bilirubin is most abundantly present in serum in its unconjugated form [28]. In a validation experiment (n = 80), we observed a strong correlation between total bilirubin and unconjugated bilirubin (Spearman’s r = 0.92, *p* < 0.001), as well as between total bilirubin and conjugated direct bilirubin (Spearman’s r = 0.82, *p* < 0.001). For the present study, we only used serum total bilirubin in keeping with other reports [29-31].

SAA was measured by a monoclonal antibody-based sandwich SAA1 enzyme-linked immunosorbent assay [24,32]. Human apo-SAA was purified from the HDL_3_ fraction of acute phase serum, linked to helix pomatia haemocyanin, and injected into Balb/c mice to produce monoclonal antihuman-SAA antibodies. The antibodies used are the capture antibody Reu.86.5, which reacts to all acute phase SAA subtypes and the coupled to horse radish peroxidase detection antibody Reu.86.1, which reacts to the major SAA1 subtype. The assay was standardized against the international standard for SAA protein (WHO code 92/680). The inter-assay CV is 7.0%. hs-CRP was assayed by nephelometry with a lower limit of 0.175 mg/l (BNII N; Dade Behring, Marburg Germany).

Plasma total cholesterol and triglycerides were assayed by routine enzymatic methods (Roche/Hitachi cat no. 11875540 and 11876023, respectively; Roche Diagnostics GmbH, Mannheim, Germany). HDL cholesterol was measured with a homogeneous enzymatic colorimetric test (Roche/Hitachi, cat no 04713214; Roche Diagnostics GmbH, Mannheim, Germany). Low density lipoprotein (LDL) cholesterol was calculated by the Friedewald formula. Glucose was analyzed with an APEC glucose analyzer (APEC Inc., Danvers, MA). HbA1c was measured by high-performance liquid chromatography (Bio-Rad, Veenendaal, the Netherlands; normal range: 4.6–6.1%). Serum aminotransferase (ALT) and aspartate aminotransferase (AST) were measured with pyridoxal phosphate activation (Merck MEGA, Darmstadt, Germany). Standardization was performed according to International Federation of Clinical Chemistry guidelines.

### Statistical analysis

SPSS 20 was used for data analysis. Results are expressed as mean ± SD or as median (interquartile range). Differences between subjects with and without MetS were determined by unpaired T tests, Mann–Whitney U tests and Chi-square tests where appropriate. Because of skewed distribution, logarithmically transformed values of bilirubin, hs-CRP, SAA and transaminases were used for linear regression analysis. Univariable relationships were calculated using Pearson correlation coefficients. Multivariable linear regression analyses were performed to determine the independent contribution of bilirubin to hs-CRP and SAA. Multivariable linear regression analyses were also carried out to determine interactions of several variables (i.e. sex, MetS, T2DM and individual MetS components) with bilirubin impacting on hs-CRP and SAA. To this end, the distribution of bilirubin was centered to its mean value by subtracting the individual value from the group mean to account for multicollinearity. Interaction terms were considered to be statistically significant at two-sided *P*-values <0.10, as recommended by Selvin [33] and by the Food and Drug Administration authorities [34]. Otherwise, the level of significance was set at two-sided *P*-values < 0.05.

## Results

Seventy-three subjects with MetS and 94 subjects without MetS were enrolled in the study (clinical characteristics shown in Table 1). Significantly more subjects with MetS had T2DM compared to subjects without MetS. Oral glucose lowering drugs (sulfonylurea and metformin, either alone or in combination; other glucose lowering drugs were not taken) were used by 41 diabetic subjects with MetS and by 17 diabetic subjects without MetS (*P* < 0.001). Twenty-seven subjects with MetS and seven subjects without MetS used anti-hypertensive medication (mostly angiotensin-converting enzyme inhibitors, angiotensin II antagonists and diuretics, either alone or in combination; *P* < 0.001). Estrogens were used by two premenopausal women with MetS and by two postmenopausal women without MetS. Age and sex distribution were not significantly different between subjects with and without MetS. Blood pressure, waist circumference, plasma glucose, insulin and HOMA-IR were higher in MetS subjects (Table 1). Plasma total cholesterol and LDL cholesterol were not different between the groups. In MetS subjects, triglycerides, hs-CRP, SAA and transaminases were increased, whereas HDL-C and bilirubin were decreased (Table 1). These differences remained statistically significant after adjustment for age, sex, and alcohol intake. In addition, hs-CRP and SAA were increased in T2DM subjects (1.74 (1.05-4.21) mg/l and 1.72 (1.13-2.57) mg/l) compared to non-diabetic subjects (1.25 (0.51-2.50) mg/l, *P* = 0.005 and 1.25 (0.82-1.98) mg/l, *P* = 0.01, respectively), but bilirubin was not significantly different between T2DM subjects (9 (7–12) μmol/l) and non-diabetic subjects 10 (7–14) μmol/l, *P* = 0.18).

**Table 1:** Characteristics in 73 subjects with metabolic syndrome (MetS) and 94 subjects without MetS

	**No MetS (n = 94)**	**MetS (n = 73)**	***P*-value**	*P*-value*
**Age (years)**	56 ± 10	58 ± 9	0.082	
**Gender (men/women)**	57/37	41/32	0.56	
**Alcohol intake (g/day)**	4.3 (0–20)	1.3 (0–8.6)	0.004	
**T2DM (yes/no)**	26/68	54/19	<0.001	
**Systolic blood pressure (mm Hg)**	131 ± 19	145 ± 18	<0.001	<0.001
**Diastolic blood pressure (mm Hg)**	81 ± 10	89 ± 9	<0.001	<0.001
**Waist circumference (cm)**	87 ± 11	104 ± 12	<0.001	<0.001
**BMI (kg/m**^**2**^**)**	25.0 ± 3.2	29.9 ± 4.5	<0.001	<0.001
**Glucose (mmol/l)**	6.1 ± 1.4	8.5 ± 2.6	<0.001	<0.001
**Insulin (mU/l)**	5.3 (4.3-7.8)	11.5 (8.6-17.0)	<0.001	<0.001
**HOMA-IR (mU × mmol)/(l**^**2 **^**× 22.5)**	1.45 (1.04-2.14)	3.96 (2.82-6.80)	<0.001	<0.001
**Total cholesterol (mmol/l)**	5.58 ± 0.99	5.56 ± 1.00	0.92	0.74
**LDL-C (mmol/l)**	3.42 ± 0.89	3.31 ± 1.00	0.46	0.59
**HDL-C(mmol/l)**	1.54 ± 0.38	1.18 ± 0.34	<0.001	<0.001
**Triglycerides (mmol/l)**	1.14 (0.85-1.56)	1.95 (1.67-2.51)	<0.001	<0.001
**Total bilirubin (μmol/l)**	11 (8–13)	9 (7–11)	0.013	0.009
**hs-CRP (mg/ml)**	1.03 (0.47-2.44)	1.97 (1.31-4.22)	<0.001	<0.001
**SAA (mg/l)**	1.25 (0.83-1.99)	1.76 (1.13-2.83)	0.002	0.027
**ALT (U/l)**	24 (20–30)	33 (24–51)	<0.001	<0.001
**AST (U/l)**	25 (22–28)	28 (24–33)	0.005	0.044

Data in mean ± SD or in median (interquartile range). ALT: serum aminotransferase; AST: serum aspartate aminotransferase; BMI: body mass index; HDL-C: high density lipoprotein cholesterol; HOMA-IR, homeostasis model assessment insulin resistance; LDL-C, low density lipoprotein cholesterol; T2DM: Type 2 diabetes mellitus. *P-value: P-value after adjustment for age, sex and alcohol intake.

**Figure 1:**
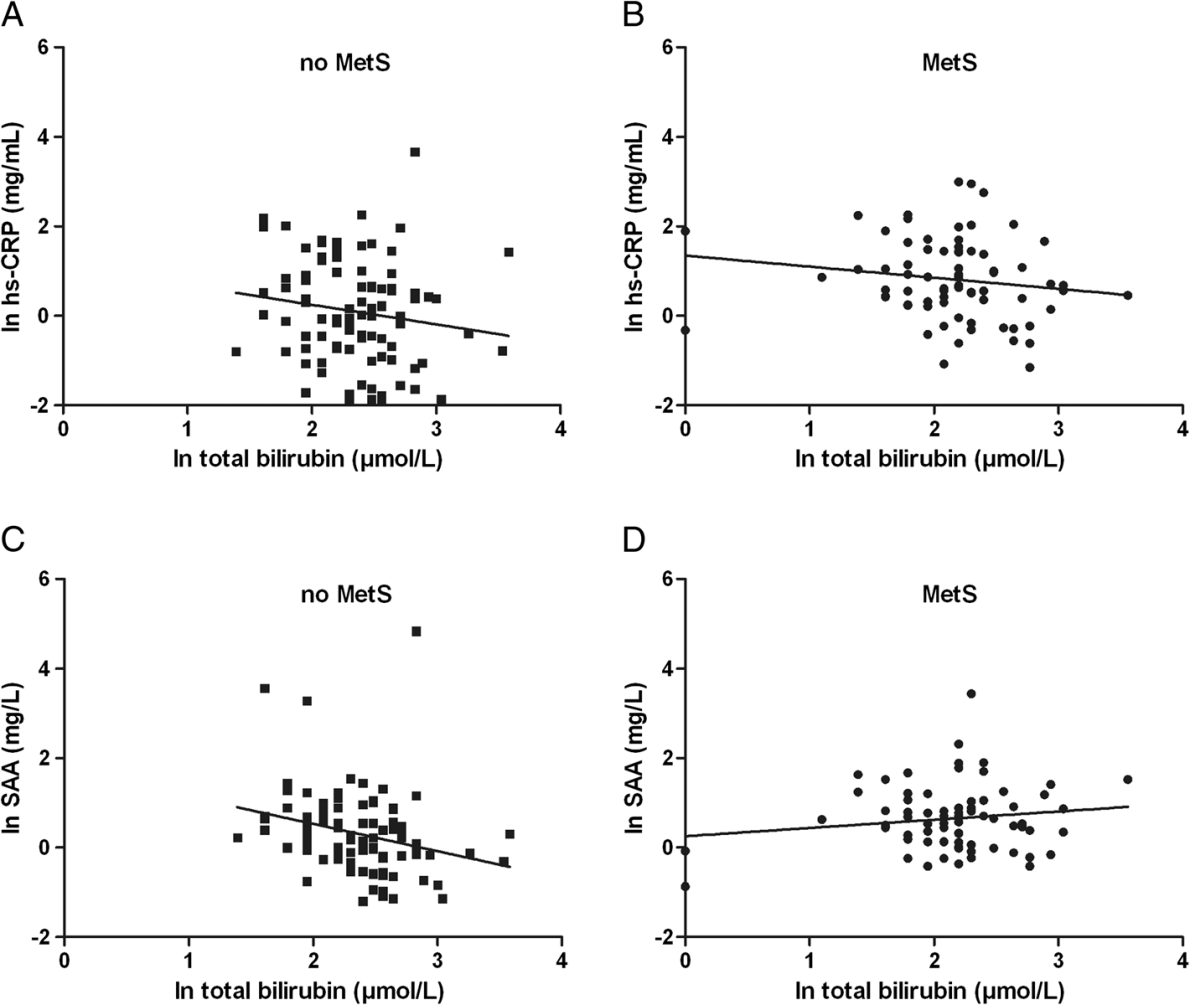
**Relationships of hs-CRP and SAA with bilirubin.** Scatterplots showing relationships of high sensitive C-reactive protein (hs-CRP) **(A, B)** and serum amyloid (SAA) **(C, D)** with bilirubin in 73 subjects with MetS **(B, D)** and 94 subjects without MetS **(A, C)**.

In the whole study population, there was an inverse correlation of hs-CRP with bilirubin, whereas similar trends towards inverse relationships were observed in subjects with and without MetS or T2DM separately (Table 2, Figure 1). The relationship of hs-CRP with bilirubin, as observed in the whole study, was not different between men and women (interaction term: β = 0.096, *P* = 0.42). This relationship was also not modified by the presence of either MetS (interaction term: β = −0.051, *P* = 0.75) or T2DM (interaction term: β = −0.010, *P* = 0.94). Despite a strong relationship between SAA and hs-CRP, SAA was not significantly correlated with bilirubin in all subjects together (Table 2). Of note, SAA was inversely related to bilirubin in subjects without MetS and in subjects without T2DM (Table 2, Figure 1). An inverse correlation of SAA with bilirubin was also observed in subjects without MetS and without T2DM, and in subjects without MetS and with T2DM, but not in subjects with MetS and without T2DM, and in subjects with both MetS and T2DM. SAA was unrelated to transaminases, but there was a positive correlation of hs-CRP with ALT in all subjects combined (r = 0.195, *P* < 0.05). There was no interaction of bilirubin with sex impacting on SAA (interaction term: β = −0.116, *P* = 0.34). Furthermore in all subjects together, bilirubin was inversely correlated with HOMA-IR (r = −0.204, *P* = 0.008). On the other hand, hs-CRP (r = 0.392, *P* < 0.001) and SAA (r = 0.213, *P* = 0.006) were positively correlated with HOMA-IR.

**Table 2:** Univariable correlations of high sensitive C-reactive protein (hs-CRP), serum amyloid A (SAA) and transaminases with bilirubin

	**hs-CRP**	**SAA**
**All subjects** (n = 167)		
hs-CRP		0.612***
Total bilirubin	−0.203**	−0.112
**No MetS** (n = 94)		
hs-CRP		0.631***
Total bilirubin	−0.154	−0.267**
**MetS** (n = 73)		
hs-CRP		0.511***
Total bilirubin	−0.155	0.144
**No T2DM** (n = 87)		
hs-CRP		0.655***
Total bilirubin	−0.216*	−0.227*
**T2DM** (n = 80)		
hs-CRP		0.522***
Total bilirubin	−0.161	0.032
**No T2DM, No MetS** (n = 68)		
hs-CRP		0.659**
Total bilirubin	−0.203	−0.267*
**T2DM , No MetS** (n = 26)		
hs-CRP		0.587**
Total bilirubin	−0.060	−0.403*
**No T2DM, MetS** (n = 19)		
hs-CRP		0.592**
Total bilirubin	−0.350	−0.045
**T2DM, MetS** (n = 54)		
hs-CRP		0.489**
Total bilirubin	−0.080	0.198

Pearson correlation coefficients are shown. ALT: serum aminotransferase; AST: serum aspartate aminotransferase; MetS: metabolic syndrome; T2DM: Type 2 diabetes mellitus. Bilirubin, hs-CRP, SAA and transaminases are logarithmically transformed. *P <0.05; **P <0.01; ***P <0.001.

We subsequently determined the extent to which the relationship of SAA with bilirubin was modified by the presence of MetS or T2DM. As shown in Table 3, the presence of MetS interacted with bilirubin on SAA (model A). The presence of T2DM also interacted with bilirubin on SAA (model C). Both interaction terms remained significant after additional adjustment for alcohol intake and transaminases (models B and D), or alternatively after adjustment for the use of oral glucose lowering drugs and anti-hypertensive medication (interaction term between MetS and bilirubin: β = 0.373, *P* = 0.003; interaction term between T2DM and bilirubin: β = 0.308, *P* = 0.019; data not shown). In an alternative analysis with HOMA-IR instead of the presence of T2DM or MetS, it was found that HOMA-IR interacted with bilirubin on SAA (Table 4, model A), also after adjustment for alcohol intake and transaminases (Table 4, model B). Furthermore, in age- and sex-adjusted analyses, it was observed that bilirubin interacted with low HDL cholesterol (interaction term: β = 0.440, *P* < 0.001), elevated triglycerides (interaction term: β = 0.289, *P* = 0.024), enlarged waist circumference (interaction term: β = 0212, *P* = 0.034), BMI (interaction term: β = 0.150, *P* = 0.048 and plasma insulin (interaction term: β = 0.190, *P* = 0.018), but not elevated glucose (interaction term: β = 0.091, *P* = 0.85) or high blood pressure (interaction term: β = −0.088, *P* = 0.45) on SAA (data not shown).

**Table 3:** Multivariable linear regression analyses on the interaction of MetS or T2DM with bilirubin on SAA

	**Model A (n = 167)**		**Model B (n = 167)**		**Model C (n = 167)**		**Model D (n = 167)**	
	**β**	***P*-value**	**β**	***P*-value**	**β**	***P*-value**	**β**	***P*-value**
**Age**	−0.033	0.67	−0.025	0.75	−0.045	0.57	−0.033	0.67
**Sex (men/women)**	−0.087	0.26	−0.101	0.21	−0.101	0.20	−0.108	0.19
**MetS (yes/no)**	0.133	0.02	0.118	0.16				
**T2DM (yes/no)**					0.142	0.075	0.085	0.32
**Total bilirubin**	−0.317	0.011	−0.311	0.013	−0.266	0.043	−0.246	0.061
Interaction MetS * total bilirubin	0.336	0.006	0.321	0.009				
Interaction T2DM * total bilirubin					0.245	0.056	0.218	0.089
**Alcohol intake**			−0.098	0.21			−0.115	0.14
**ALT**			0.098	0.28			0.093	0.32
**AST**			0.059	0.48			0.072	0.40

ALT: aminotransferase; AST: aspartate aminotransferase; MetS: metabolic syndrome; SAA: serum amyloid A; T2DM: type 2 diabetes mellitus; β: standardized regression coefficient.

**Model A:** includes age, sex, bilirubin, MetS, T2DM and interaction of MetS with bilirubin.

**Model B:** additionally includes alcohol intake and transaminases.

**Model C:** model including age, sex, bilirubin, MetS, T2DM and interaction of T2DM with bilirubin.

**Model D:** additionally includes alcohol intake and transaminases.

**Table 4:** Multivariable linear regression analyses on the interaction of HOMA with bilirubin on SAA

	**Model A (n = 167)**		**Model B (n = 167)**	
	**β**	**P-value**	**β**	**P-value**
**Age**	−0.034	0.66	−0.027	0.73
**Sex**	−0.099	0.20	−0.097	0.23
**Bilirubin**	−0.097	0.24	−0.099	0.23
**HOMA-IR**	−0.572	0.094	−0.551	0.11
Bilirubin * HOMA-IR	0.790	0.020	0.720	0.035
**Alcohol**			−0.109	0.16
**ALT**			0.038	0.70
**AST**			0.070	0.41

ALT: alanine aminotransferase; AST: aspartate aminotransferase; HOMA: homeostasis model assessment-insulin resistance; β: standardized regression coefficient.

**Model A:** includes age, sex, bilirubin, HOMA and the interaction between bilirubin and HOMA.

**Model B:** additionally includes alcohol intake and transaminases.

## Discussion

The current study demonstrates that hs-CRP is inversely related to bilirubin irrespective of the presence of MetS or T2DM. In view of lower bilirubin and higher hs-CRP levels in MetS our findings support the possibility that lower bilirubin could be implicated in enhanced low-grade systemic inflammation in MetS. In univariable analysis, SAA was inversely correlated with bilirubin in subjects without MetS, but not in subjects with MetS. Remarkably, effect modification of the relationship of SAA with bilirubin was observed in the context of MetS, T2DM and insulin resistance, despite expected robust correlations between hs-CRP and SAA levels, which were present in all subject categories.

Lower bilirubin levels in MetS, and the inverse relationship of bilirubin with HOMA-IR extend other studies that reported on such inverse relationships of bilirubin with abdominal obesity [35], insulin resistance [36] and diabetes [3,37]. In a large Korean cohort, higher bilirubin levels were also found to be associated with a low prevalence of MetS [5]. In agreement with previous studies, we also found an inverse correlation of hs-CRP with bilirubin [9,10], which is clearly in line with allegedly anti-inflammatory properties of bilirubin. Bilirubin is known to interfere with expression of adhesion molecules VCAM-1 and ICAM-1 [1], with activity of the complement system [38] and with T cell differentiation [39]. Hence, it is conceivable that anti-inflammatory and anti-oxidative effects of bilirubin may both contribute to less low-grade chronic inflammation in MetS [3,15,18,25,26,40]. The only study available so far showed that SAA tended to be lower in subjects with isolated hyperbilirubinemia due to Gilbert syndrome compared to healthy individuals [17], suggesting that high bilirubin levels could be implicated in lower SAA levels. In the current study, the relationship of SAA with bilirubin was modified by insulin and HOMA-IR, but not by elevated glucose. These results, therefore, raise the possibility that (factors which are closely associated with) insulin resistance, rather than hyperglycemia *per se* may explain the absence of a correlation of SAA with bilirubin in insulin resistant individuals.

Alterations in compositional characteristics of HDL in MetS are likely to contribute to impaired HDL anti-oxidative function, as well as to increased systemic oxidative stress [4,19]. MetS is also featured by decreased levels of the anti-oxidant, paraoxonase-1 [41]. In this regard, it is important that SAA, which is carried on HDL particles, is able to displace apolipoprotein A-I and paraoxonase-1 from HDL particles, thereby impairing the ability of HDL to protect LDL from oxidative modification [19,20]. In agreement, we recently observed an inverse relationship of HDL anti-oxidative capacity with SAA in healthy subjects [18]. Of note, such a relationship was found to be absent in MetS [18], in line with the lack of association of SAA with bilirubin in MetS, as observed in the current report. Furthermore, we documented that the positive relationship of total plasma apolipoprotein E with paraoxonase-1 is absent in MetS [42]. Taken together, these previous [18,42] and the present findings would raise the possibility that MetS or insulin resistance may elicit abnormalities in HDL anti-oxidative function, which could mask a relationship of SAA with bilirubin. Indeed, it is noteworthy that of the individual MetS components the strongest effect modification of bilirubin on SAA was observed for HDL cholesterol. However, we did not document oxidative stress in our study population, and consider the present findings regarding the lack of relationship of bilirubin with SAA in subjects with MetS to be hypothesis generating. More work is needed to delineate the extent to which bilirubin may modify the functional properties of HDL and the proposed role of SAA therein in order to better understand the protective role of bilirubin in cardiovascular disease [7,8].

Several other methodological issues and limitations of our study need to be considered. Since we carried out a cross-sectional study, cause-effect relationships concerning the relationship of hs-CRP and SAA with bilirubin cannot be ascertained with certainty. Furthermore, data with respect to physical fitness and a detailed diet history were not available. The inclusion of T2DM subjects in the present study allowed us to discern that the relationship of hs-CRP with bilirubin was not relevantly modified by the presence of either MetS or T2DM. In addition, effect modification of bilirubin on SAA could be demonstrated with respect to the presence of MetS, T2DM and the degree of insulin resistance.

In conclusion, this study suggests that lower bilirubin may confer enhanced low-grade systemic inflammation, as evidenced by higher hs-CRP levels, irrespective of the presence of MetS. In contrast, the inverse relationship of SAA with bilirubin was confined to subjects without MetS, possibly consequent to MetS-associated abnormalities in HDL characteristics.

## Abbreviations

BMI: Body mass index; HbA1c: Glycated hemoglobin; HDL: High-density lipoprotein; HOMA-IR: Homeostasis model assessment-insulin resistance; hs-CRP: High sensitive C-reactive protein; MetS: Metabolic syndrome; NCEP-ATPIII: National cholesterol education program - adult treatment panel III; SAA: Serum amyloid A; T2DM: Type 2 diabetes mellitus.

## Competing interests

The authors declare that they have no competing interests.

## Authors’ contributions

PD analyzed the data and drafted the manuscript; SB participated in intellectual contributions and manuscript preparation; RD initiated the study, performed study planning, supervised data collection and participated in subject care and manuscript preparation. All authors read and approved the final manuscript.

## References

[B1] Mazzone GL Rigato I Ostrow JD Bossi F Bortoluzzi A Sukowati CH Tedesco F Tiribelli C Bilirubin inhibits the TNFalpha-related induction of three endothelial adhesion molecules Biochem Biophys Res Commun 2009 386 2 338 344 10.1016/j.bbrc.2009.06.029 19523446

[B2] Abraham NG Kappas A Heme oxygenase and the cardiovascular-renal system Free Radic Biol Med 2005 39 1 1 25 10.1016/j.freeradbiomed.2005.03.010 15925276

[B3] Vitek L The role of bilirubin in diabetes, metabolic syndrome, and cardiovascular diseases Front Pharmacol 2012 3 55 22493581 10.3389/fphar.2012.00055 PMC3318228

[B4] Hansel B Giral P Nobecourt E Chantepie S Bruckert E Chapman MJ Kontush A Metabolic syndrome is associated with elevated oxidative stress and dysfunctional dense high-density lipoprotein particles displaying impaired antioxidative activity J Clin Endocrinol Metab 2004 89 10 4963 4971 10.1210/jc.2004-0305 15472192

[B5] Choi SH Yun KE Choi HJ Relationships between serum total bilirubin levels and metabolic syndrome in Korean adults Nutr Metab Cardiovasc Dis 2013 23 1 31 37 10.1016/j.numecd.2011.03.001 21703835

[B6] Robertson RP Harmon JS Diabetes, glucose toxicity, and oxidative stress: A case of double jeopardy for the pancreatic islet beta cell Free Radic Biol Med 2006 41 2 177 184 10.1016/j.freeradbiomed.2005.04.030 16814095

[B7] Kim KM Kim BT Park SB Cho DY Je SH Kim KN Serum total bilirubin concentration is inversely correlated with Framingham risk score in Koreans Arch Med Res 2012 43 4 288 293 10.1016/j.arcmed.2012.05.003 22595233

[B8] Kang SJ Kim D Park HE Chung GE Choi SH Choi SY Lee W Kim JS Cho SH Elevated serum bilirubin levels are inversely associated with coronary artery atherosclerosis Atherosclerosis 2013 230 2 242 248 10.1016/j.atherosclerosis.2013.06.021 24075751

[B9] Vitek L Novotny L Sperl M Holaj R Spacil J The inverse association of elevated serum bilirubin levels with subclinical carotid atherosclerosis Cerebrovasc Dis 2006 21 5–6 408 414 16534198 10.1159/000091966

[B10] Yoshino S Hamasaki S Ishida S Kataoka T Yoshikawa A Oketani N Saihara K Okui H Shinsato T Ichiki H Kubozono T Kuwahata S Fujita S Kanda D Nakazaki M Miyata M Tei C Relationship between bilirubin concentration, coronary endothelial function, and inflammatory stress in overweight patients J Atheroscler Thromb 2011 18 5 403 412 10.5551/jat.6346 21350306

[B11] Dullaart RP Kappelle PJ De Vries R Lower carotid intima media thickness is predicted by higher serum bilirubin in both non-diabetic and Type 2 diabetic subjects Clin Chim Acta 2012 414 161 165 23010146 10.1016/j.cca.2012.08.029

[B12] Novotny L Vitek L Inverse relationship between serum bilirubin and atherosclerosis in men: a meta-analysis of published studies Exp Biol Med (Maywood) 2003 228 5 568 571 12709588 10.1177/15353702-0322805-29

[B13] Chan KH O’Connell RL Sullivan DR Hoffmann LS Rajamani K Whiting M Donoghoe MW Vanhala M Hamer A Yu B Stocker R Ng MK Keech AC FIELD study investigators: plasma total Bilirubin levels predict amputation events in type 2 diabetes mellitus: the Fenofibrate Intervention and Event Lowering in Diabetes (FIELD) study Diabetologia 2013 56 4 724 736 10.1007/s00125-012-2818-4 23322233

[B14] Ajja R Lee DC Sui X Church TS Steven NB Usefulness of serum Bilirubin and cardiorespiratory fitness as predictors of mortality in men Am J Cardiol 2011 108 10 1438 1442 10.1016/j.amjcard.2011.06.067 21864819 PMC3206143

[B15] Vitek L Schwertner HA The heme catabolic pathway and its protective effects on oxidative stress-mediated diseases Adv Clin Chem 2007 43 1 57 17249379 10.1016/s0065-2423(06)43001-8

[B16] Hwang HJ Lee SW Kim SH Relationship between Bilirubin and C-reactive protein Clin Chem Lab Med 2011 49 11 1823 1828 21726166 10.1515/CCLM.2011.662

[B17] Wallner M Marculescu R Doberer D Wolzt M Wagner O Vitek L Bulmer AC Wagner KH Protection from age-related increase in lipid biomarkers and inflammation contributes to cardiovascular protection in Gilbert’s syndrome Clin Sci (Lond) 2013 125 5 257 264 10.1042/CS20120661 23566065

[B18] Dullaart RP De Boer JF Annema W Tietge UJ The inverse relation of HDL anti-oxidative functionality with serum amyloid a is lost in metabolic syndrome subjects Obesity (Silver Spring) 2013 21 2 361 366 10.1002/oby.20058 23404653

[B19] Kontush A Chapman MJ Functionally defective high-density lipoprotein: a new therapeutic target at the crossroads of dyslipidemia, inflammation, and atherosclerosis Pharmacol Rev 2006 58 3 342 374 10.1124/pr.58.3.1 16968945

[B20] Nofer J Serum amyloid A concentration may reflect HDL functionality Clin Lipidolin press

[B21] Ridker PM Hennekens CH Buring JE Rifai N C-reactive protein and other markers of inflammation in the prediction of cardiovascular disease in women N Engl J Med 2000 342 12 836 843 10.1056/NEJM200003233421202 10733371

[B22] Danesh J Whincup P Walker M Lennon L Thomson A Appleby P Gallimore JR Pepys MB Low grade inflammation and coronary heart disease: prospective study and updated meta-analyses BMJ 2000 321 7255 199 204 10.1136/bmj.321.7255.199 10903648 PMC27435

[B23] Johnson BD Kip KE Marroquin OC Ridker PM Kelsey SF Shaw LJ Pepine CJ Sharaf B Bairey Merz CN Sopko G Olson MB Reis SE National Heart, Lung, and Blood Institute: Serum amyloid A as a predictor of coronary artery disease and cardiovascular outcome in women: the National Heart, Lung, and Blood Institute-Sponsored Women’s Ischemia Syndrome Evaluation (WISE) Circulation 2004 109 6 726 732 10.1161/01.CIR.0000115516.54550.B1 14970107

[B24] Kappelle PJ Bijzet J Hazenberg BP Dullaart RP Lower serum paraoxonase-1 activity is related to higher serum amyloid a levels in metabolic syndrome Arch Med Res 2011 42 3 219 225 10.1016/j.arcmed.2011.05.002 21722818

[B25] Jylhava J Haarala A Eklund C Pertovaara M Kahonen M Hutri-Kahonen N Levula M Lehtimaki T Huupponen R Jula A Juonala M Viikari J Raitakari O Hurme M Serum amyloid a is independently associated with metabolic risk factors but not with early atherosclerosis: the cardiovascular risk in young Finns study J Intern Med 2009 266 3 286 295 10.1111/j.1365-2796.2009.02120.x 19702793

[B26] Den Engelsen C Koekkoek PS Gorter KJ Van den Donk M Salome PL Rutten GE High-sensitivity C-reactive protein to detect metabolic syndrome in a centrally obese population: a cross-sectional analysis Cardiovasc Diabetol 2012 11 25 2840 11-25 10.1186/1475-2840-11-25 22417460 PMC3359236

[B27] Grundy SM Cleeman JI Daniels SR Donato KA Eckel RH Franklin BA Gordon DJ Krauss RM Savage PJ Smith SC Jr Spertus JA Costa F American Heart Association, National Heart, Lung, and Blood Institute: Diagnosis and management of the metabolic syndrome: an American Heart Association/National Heart, Lung, and Blood Institute Scientific Statement Circulation 2005 112 17 2735 2752 10.1161/CIRCULATIONAHA.105.169404 16157765

[B28] Tisdale WA Klatskin G Kinsella ED The significance of the direct-reacting fraction of serum Bilirubin in hemolytic jaundice Am J Med 1959 26 2 214 227 10.1016/0002-9343(59)90310-9 13617278

[B29] Zelle DM Deetman N Alkhalaf A Navis G Bakker SJ Support for a protective effect of Bilirubin on diabetic nephropathy in humans Kidney Int 2011 79 6 686 7 21358656 10.1038/ki.2010.503

[B30] Katsiki N Karagiannis A Mikhailidis DP Diabetes, bilirubin and amputations: is there a link? Diabetologia 2013 56 4 683 685 10.1007/s00125-013-2840-1 23354127

[B31] Deetman PE Zelle DM Van der Heide JJ H Navis GJ Gans RO Bakker SJ Plasma bilirubin and late graft failure in renal transplant recipients Transpl Int 2012 25 8 876 881 10.1111/j.1432-2277.2012.01515.x 22716194

[B32] Hazenberg BP Limburg PC Bijzet J Van Rijswijk MH A quantitative method for detecting deposits of amyloid A protein in aspirated fat tissue of patients with arthritis Ann Rheum Dis 1999 58 2 96 102 10.1136/ard.58.2.96 10343524 PMC1752828

[B33] Selvin S Statistical Analysis of Epidemiological Data 1996 New York: Oxford University Press

[B34] Lu M Lyden PD Brott TG Hamilton S Broderick JP Grotta JC Beyond subgroup analysis: improving the clinical interpretation of treatment effects in stroke research J Neurosci Methods 2005 143 2 209 216 10.1016/j.jneumeth.2004.10.002 15814153

[B35] Jenko-Praznikar Z Petelin A Jurdana M Ziberna L Serum bilirubin levels are lower in overweight asymptomatic middle-aged adults: An early indicator of metabolic syndrome? Metabolism 2013 62 7 976 985 10.1016/j.metabol.2013.01.011 23414908

[B36] Lin LY Kuo HK Hwang JJ Lai LP Chiang FT Tseng CD Lin JL Serum bilirubin is inversely associated with insulin resistance and metabolic syndrome among children and adolescents Atherosclerosis 2009 203 2 563 568 10.1016/j.atherosclerosis.2008.07.021 18775539

[B37] Ohnaka K Kono S Inoguchi T Yin G Morita M Adachi M Kawate H Takayanagi R Inverse associations of serum bilirubin with high sensitivity C-reactive protein, glycated hemoglobin, and prevalence of type 2 diabetes in middle-aged and elderly Japanese men and women Diabetes Res Clin Pract 2010 88 1 103 110 10.1016/j.diabres.2009.12.022 20083320

[B38] Basiglio CL Arriaga SM Pelusa F Almara AM Kapitulnik J Mottino AD Complement activation and disease: protective effects of hyperbilirubinaemia Clin Sci (Lond) 2009 118 2 99 113 10.1042/CS20080540 19807696

[B39] Rocuts F Zhang X Yan J Yue Y Thomas M Bach FH Czismadia E Wang H Bilirubin promotes de novo generation of T regulatory cells Cell Transplant 2010 19 4 443 451 10.3727/096368909X484680 20021735

[B40] Rutter MK Meigs JB Sullivan LM D’Agostino RBS Wilson PW C-reactive protein, the metabolic syndrome, and prediction of cardiovascular events in the Framingham Offspring Study Circulation 2004 110 4 380 385 10.1161/01.CIR.0000136581.59584.0E 15262834

[B41] Garin MC Kalix B Morabia A James RW Small, dense lipoprotein particles and reduced paraoxonase-1 in patients with the metabolic syndrome J Clin Endocrinol Metab 2005 90 4 2264 2269 10.1210/jc.2004-1295 15687341

[B42] Dullaart RP Kwakernaak AJ Dallinga-Thie GM The positive relationship of serum paraoxonase-1 activity with apolipoprotein E is abrogated in metabolic syndrome Atherosclerosis 2013 230 1 6 11 10.1016/j.atherosclerosis.2013.06.019 23958245

